# Advancements in bioengineering for descemet membrane endothelial keratoplasty (DMEK)

**DOI:** 10.1038/s41536-025-00396-0

**Published:** 2025-02-14

**Authors:** Sarah Barbara Zwingelberg, Gizem Karabiyik, Paul Gehle, Melanie von Brandenstein, Sabina Eibichova, Christian Lotz, Florian Groeber-Becker, Daniel Kampik, Ula Jurkunas, Gerd Geerling, Gregor Lang

**Affiliations:** 1 Department of Ophthalmology, University Hospital of Duesseldorf, Duesseldorf, Germany; 2 Department of Functional Materials in Medicine and Dentistry, University Hospital Würzburg, Würzburg, Germany; 3 Department of Urology, University of Cologne, Faculty of Medicine and University Hospital of Cologne, Cologne, Germany; 4 University Hospital Würzburg, Department of Tissue Engineering and Regenerative Medicine, Würzburg, Germany; 5 Fraunhofer Institute for Silicate Research ISC Translational Center Regenerative Therapies, Würzburg, Germany; 6 Department of Ophthalmology, University Hospital Würzburg, Würzburg, Germany; 7 Department of Ophthalmology, Harvard Medical School, Schepens Eye Institute, Boston, MA USA

**Keywords:** Tissues, Corneal diseases

## Abstract

Corneal diseases are the third leading cause of blindness worldwide. Descemet’s Membrane Endothelial Keratoplasty (DMEK) is the preferred surgical technique for treating corneal endothelial disorders, relying heavily on high-quality donor tissue. However, the scarcity of suitable donor tissue and the sensitivity of endothelial cells remain significant challenges. This review explores the current state of DMEK, focusing on advancements in tissue engineering as a promising solution to improve outcomes and address donor limitations.

## Introduction

Corneal diseases are the third leading cause of blindness worldwide^1^. The medical relevance of artificial corneas has gained importance, particularly due to the SARS-CoV-2 pandemic. A multicenter study from 26 European countries, involving 110 cornea banks, highlighted a rapidly growing donor shortage in 2020, showing an average monthly decline of 38%, 68%, and 41% in donor numbers from February 2018 to May 2020^2^. This situation emphasizes the urgent need to replace the apparent donor shortage with artificial corneal transplants and to intensify research in this field. The global situation is even more dire, especially in countries lacking regulated access to corneal transplantation.

The reasons for corneal transplantation are diverse, including acute and chronic infections, previous trauma, degenerative diseases, and corneal dystrophies like Fuchs endothelial corneal dystrophy (FECD), which affects the corneal endothelium. The endothelium is a monolayer of specialized cells located at the back of the eye, essential for maintaining corneal transparency by regulating stromal hydration and pumping excess fluid out of the cornea. Dysfunction of the corneal endothelium can lead to visual impairment and blindness, necessitating surgical intervention.

FECD is characterized by a progressive deterioration of the corneal endothelium, resulting in vision impairment and discomfort. As one of the most common causes of corneal edema and subsequent vision loss, FECD poses significant challenges to affected individuals. It is a complex and heterogeneous genetic disease where the interplay of genetic and environmental factors leads to oxidative stress, autophagy (including mitophagy), unfolded protein response, mitochondrial dysfunction, and ultimately the progressive decline of corneal endothelial cells (CECs) through cellular apoptosis^3^. FECD manifests variations in the size (polymegathism) and shape (pleomorphism) of CECs, decreased endothelial cell density (ECD), and the formation of extracellular matrix (ECM) outgrowths known as guttae^4,5^.

Family history is a significant risk factor, indicating a genetic predisposition to the condition. The relevant genes have been mapped and identified, with specific mutations recognized^6^. Two types of FECD are noted: early-onset FECD, which manifests in the first decade of life, and late-onset FECD, which occurs after age 40 and has a higher incidence compared to the early-onset form. Both forms are associated with genetic changes, with the early-onset form predominantly linked to alterations in the *COL8A* gene on chromosome 1p34.3-32.3. The genetics of late-onset FECD appear more complex and varied, typically involving mutations in the transcription factor 4 (TCF4) gene on chromosome 18, characterized by an expansion of a CTG triplet repeat. Additionally, other genes such as transcription factor 8 (TCF8), ATP/GTP binding protein like 1 (*AGBL1*), lipoxygenase homology domain 1 (*LOXHD1*), SLC transporter *SLC4A11*, and transforming growth factor-beta induced (*TGFBI*) are also implicated^6,7^. Epigenetic factors are considered additional pathogenetic mechanisms for both types of FECD^8,9^.

While the exact cause of FECD remains elusive, both genetic and environmental factors—such as UV-A light exposure, smoking, obesity, and diabetes—are believed to contribute to its development through interactive influences on epigenetic changes^10^.

Smoking has detrimental effects on the development of FECD, increasing free radicals and decreasing antioxidants in the blood and ocular tissues. Consequently, smokers’ eyes are at higher risk for oxidative stress and free radical damage, leading to increased apoptosis of endothelial cells and a higher prevalence of corneal guttae, as well as more severe Krachmer grading in smokers^11–18^.

**Fig. 1:**
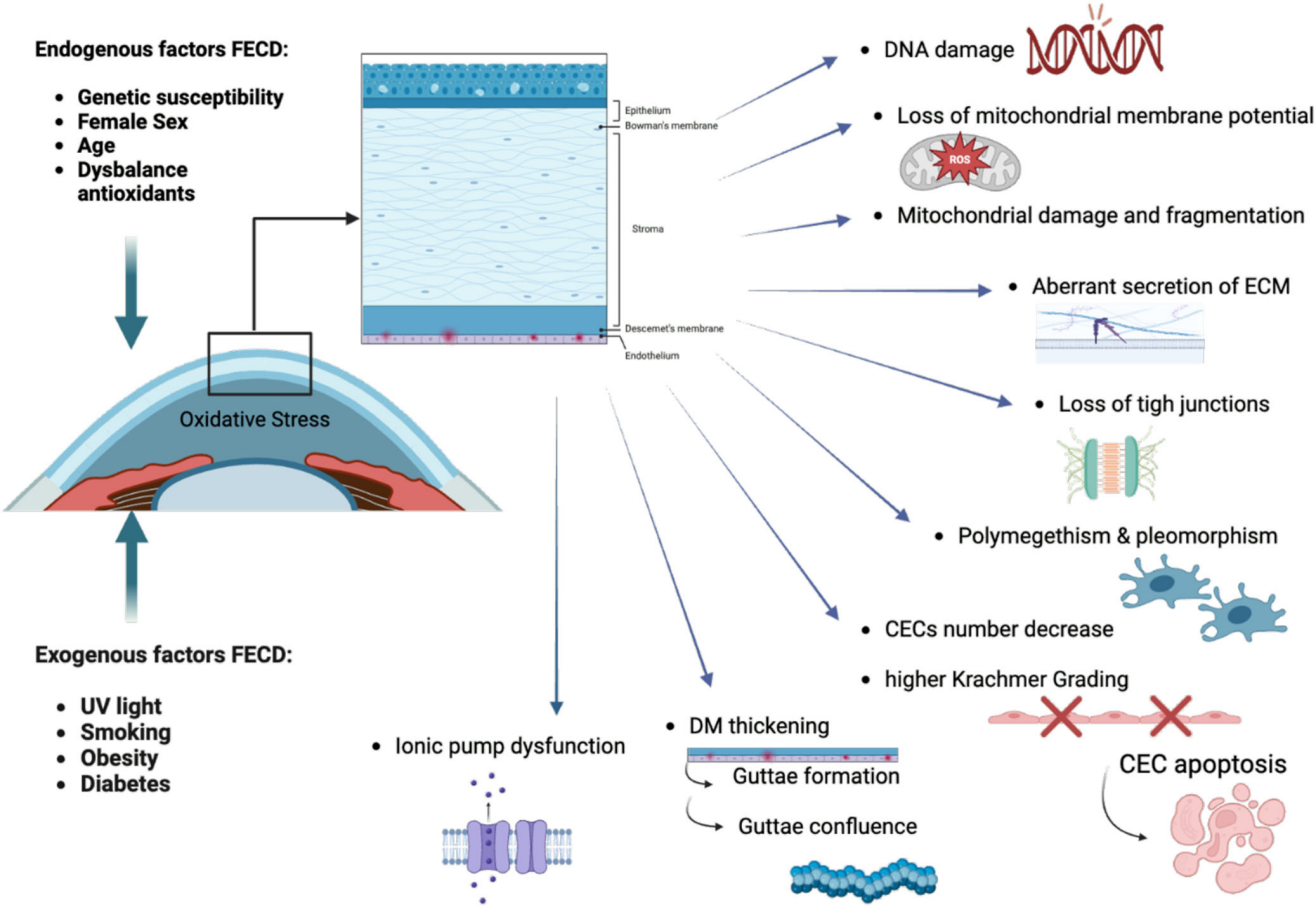
Schematic representation of the multifactorial genesis of Fuchs endothelial corneal dystrophy (FECD). Created by S. Zwingelberg with Biorender.com.

Obesity, defined by a Body Mass Index (BMI) ≥ 30, is also associated with an earlier onset of FECD. The comorbidity of diabetes mellitus correlates with higher Krachmer grades (Fig. 1)^19–27^.

FECD typically manifests in the fifth or sixth decade of life and occurs more frequently in women, with a ratio of 3:1 to 4:1, and it tends to progress slowly over time^10,28–30^. The early stages of FECD may be asymptomatic, with symptoms becoming more pronounced as the condition progresses. Common signs and symptoms of FECD include blurred or cloudy vision, particularly in the morning upon waking; increased sensitivity to glare; halos or starbursts around lights; eye discomfort or pain, often exacerbated by bright light or windy conditions; decreased visual acuity, even with corrective lenses; and corneal swelling (edema), leading to a thickened or hazy appearance of the cornea. Persistent corneal edema causes the death of keratocytes in the stroma and subepithelial fibrosis, resulting in an irregular anterior cornea and loss of vision, which is observed as a fibrillar layer on slit-lamp microscopy, first described by Matthaei et al. in 2021^31,32^. The diagnosis of FECD typically involves a comprehensive eye examination by an ophthalmologist.

Key diagnostic tests should include best-corrected visual acuity assessment, a glare vision test, a slit-lamp examination to evaluate the corneal endothelium and detect characteristic findings such as corneal guttae (tiny excrescences on the inner surface of the cornea), measurement of corneal pachymetry to assess corneal thickness and signs of edema, and specular microscopy to examine the density and morphology of corneal endothelial cells.

While there is no cure for FECD, several treatment options aim to manage its symptoms and slow disease progression. These may include conservative therapy with hypertonic saline drops or ointments to reduce corneal edema and alleviate symptoms. However, corneal transplantation is currently the only effective treatment option for FECD patients in the form of endothelial lamellar keratoplasty, especially Descemet Membrane Endothelial Keratoplasty (DMEK), which replaces the damaged corneal endothelium with healthy donor tissue^33–35^. In addition to FECD, DMEK is indicated for the following conditions of endothelial dysfunction:

- Bullous Keratopathy: This condition results from endothelial cell loss due to surgical interventions (e.g., cataract surgery) or pre-existing conditions like Fuchs’ corneal dystrophy. It causes painful blisters on the corneal surface, progressively leading to vision loss over time^36^.
- Graft Rejection Following Previous Corneal Transplants (e.g., PK failure): When a previous corneal graft, such as penetrating keratoplasty (PK), fails due to an immune response damaging the endothelial cells, DMEK is a feasible alternative (compare Fig. 3). The long-term success of DMEK in patients with failed PKs has been well-documented and shows good results over time^37,38^.
- Iridocorneal Endothelial Syndrome (ICE Syndrome): This rare disorder is characterized by abnormalities in the corneal endothelial cells, deformation of the iridocorneal angle, and abnormalities in the iris stroma, which lead to corneal edema and glaucoma. Due to the severe irregularities of the anterior segment in ICE syndrome, corneal decompensation and glaucoma pose significant challenges in surgical treatment. Patients undergoing PK for ICE syndrome have exhibited high incidences of graft rejection and endothelial failure. However, treating ICE syndrome with DMEK has resulted in higher visual acuity and lower rejection rates compared to PK and DSEK^36,39,40^. Although long-term data on DMEK in ICE syndrome remain limited, early results show promising improvements in visual acuity, positioning DMEK as a viable and effective treatment option^41,42^.

Other indications for DMEK include endothelial dysfunction following trauma, corneal decompensation due to glaucoma, and herpes simplex virus endotheliitis. Short- or long-term endothelial damage resulting from scarring, glaucoma, or recurrent viral infections can threaten vision over time. Historically, PK was the primary treatment option for these cases. However, recent outcomes suggest that DMEK is an effective surgical approach for corneal endothelial decompensation secondary to scarring, glaucoma, or recurrent herpes simplex infections^43,44^. DMEK offers significant advantages in many corneal endothelial disorders, including faster visual recovery, better long-term outcomes, lower immune rejection rates, and reduced refractive errors compared to older techniques like DSEK/DSAEK or PK^45^.

### Endothelial Keratoplasty

There are two primary forms of endothelial keratoplasty used to treat corneal endothelial dysfunction: Descemet Membrane Endothelial Keratoplasty (DMEK) and Descemet Stripping (Automated) Endothelial Keratoplasty (DS(A)EK).

### Descemet Membrane Endothelial Keratoplasty (DMEK)

DMEK has emerged as a revolutionary surgical technique, offering a good opportunity for patients with conditions like FECD and endothelial failure. It represents a paradigm shift in corneal transplantation, promising superior visual outcomes, faster recovery, and reduced risk of rejection due to the selective lamellar transplantation of the Descemet membrane and endothelial cells from a donor cornea to a recipient eye^46–50^.

Unlike traditional full-thickness corneal transplants, DMEK preserves the recipient’s healthy corneal layers, leading to better visual outcomes and a reduced risk of postoperative complications, such as donor graft rejection.

### Descemet Stripping (Automated) Endothelial Keratoplasty (DS(A)EK)

**Fig. 2:**
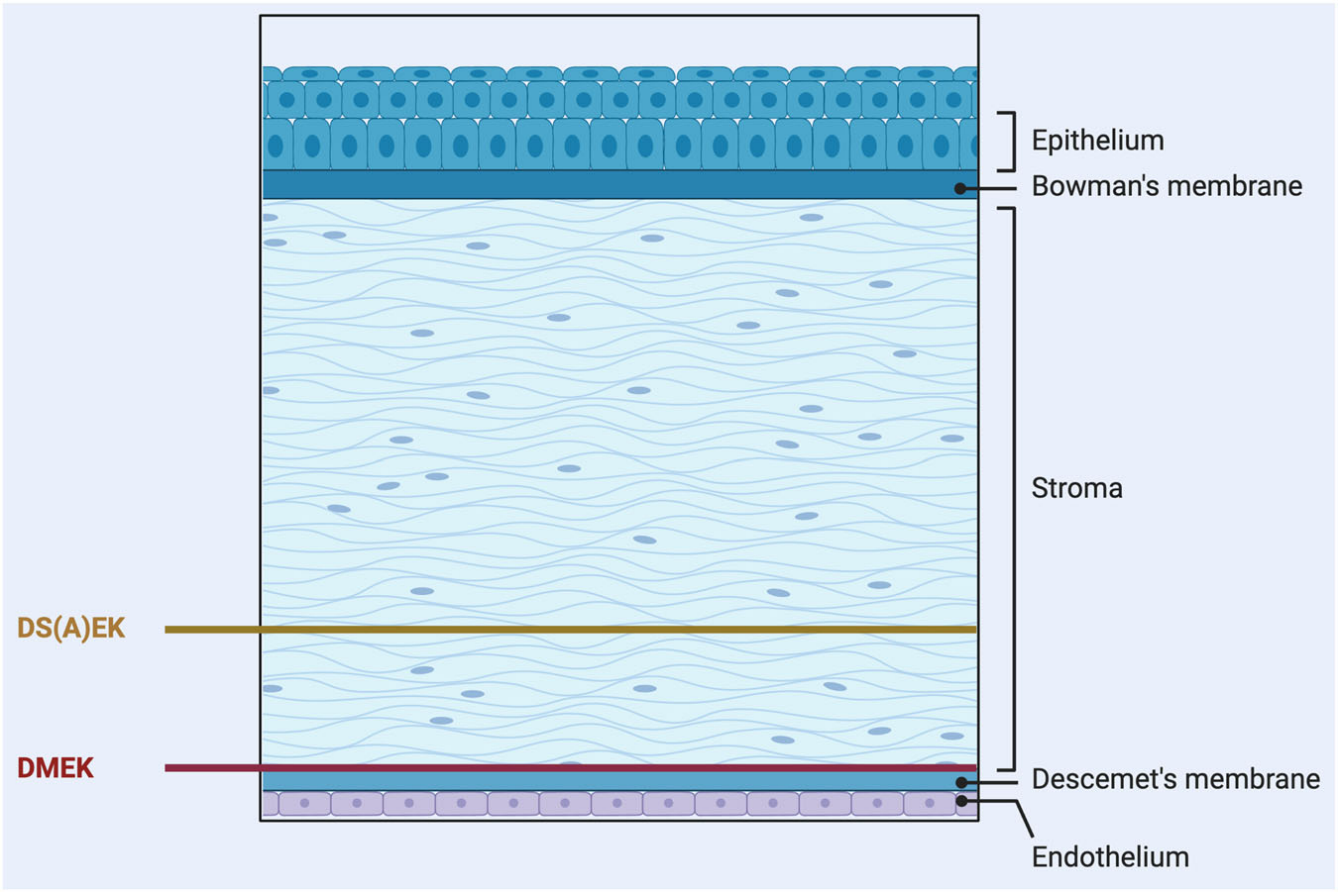
Schematic overview of Descemet Membrane Endothelial Keratoplasty (DMEK) procedure versus Descemet Stripping (Automated) Endothelial Keratoplasty (DS(A)EK). Created by S. Zwingelberg with Biorender.com.

DS(A)EK involves transplanting both the Descemet membrane and a thin layer of the stroma along with the endothelial cells using an automated instrument known as a microkeratome (Fig. 2). While DS(A)EK is technically easier to perform and thus in some countries still more widely practiced, the thicker graft may results in slower and slightly lower visual recovery and a higher likelihood of refractive changes compared to DMEK, even in eyes with retinal comorbidities.

Both procedures are considered less invasive and have fewer complications than traditional full-thickness corneal transplants. However, DMEK is increasingly favored due to its superior visual outcomes and lower rejection rates.

### Advantages of DMEK- versus DS(A)EK- concerning the surgical procedure

Although preparing a graft for DMEK is more challenging than for DSEK/DSAEK, it offers faster and more complete visual recovery by excluding corneal stroma, which significantly reduces the risk of graft rejection and scar formation at a stromal interface level after surgery. This allows patients after DMEK to return to their daily activities more quickly^51,52^.

Another advantage of DMEK is that patients require less topical corticosteroid use to prevent rejection compared to PK and DSEK, thereby reducing the risk of steroid-induced increases in intraocular pressure and secondary glaucoma^53^. Additionally, DMEK promotes the efficient and judicious use of a single donor cornea, allowing one cornea to be utilized for both DMEK and DALK, which is a critical factor given the worldwide shortage of corneal donors (54). These benefits make the DMEK method advantageous over other endothelial keratoplasty (EK) techniques, contributing to its rapid increase in use and growing attention recently^48,54^.

In a previous experimental study, 15 patients with endothelial dysfunction (30 eyes total) underwent DSAEK in one eye and DMEK in the fellow eye. The study aimed to compare DSAEK and DMEK from various perspectives and assess patient satisfaction. Observations indicated that eyes undergoing DMEK tended to experience faster improvements in vision compared to those that underwent DSAEK. Although the loss of endothelial cells and the ultimate visual outcomes were recorded as similar in both procedures, most patients preferred or would recommend the DMEK technique over DSAEK^54^.

### Advantage of DMEK procedure concerning immunological reactions

**Fig. 3:**
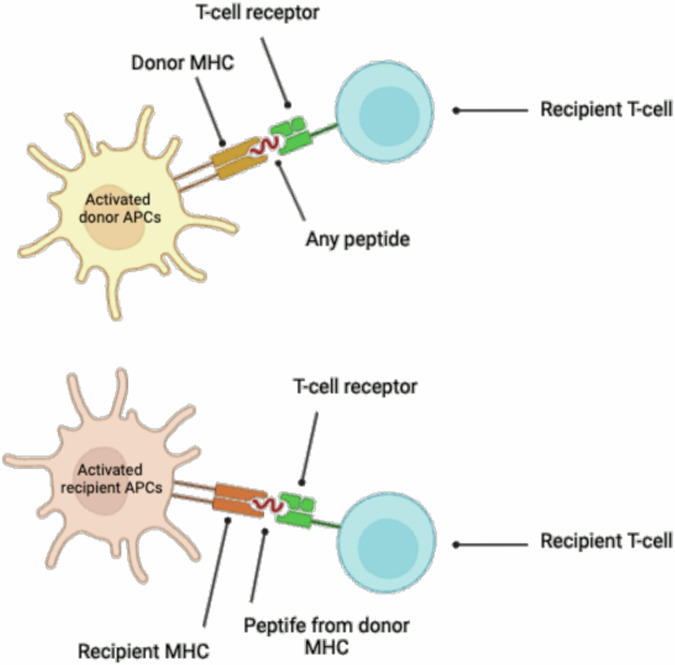
Schematic overview of direct and indirect alloantigen recognition in corneal transplantation. Created by S. Zwingelberg with Biorender.com.

In the field of transplant immunology, both direct and indirect alloantigen recognition play pivotal roles (compare Fig. 3).

- Direct alloantigen recognition occurs when donor antigen-presenting cells (APCs) present intact donor major histocompatibility complex (MHC) molecules to recipient T cells. This direct presentation activates the recipient’s T cells against the donor antigens.
- Indirect alloantigen recognition involves recipient APCs presenting processed donor antigens, including donor MHC peptides, on recipient MHC molecules to recipient T cells. This indirect pathway can also trigger an immune response against the donor graft.

**Fig. 4:**
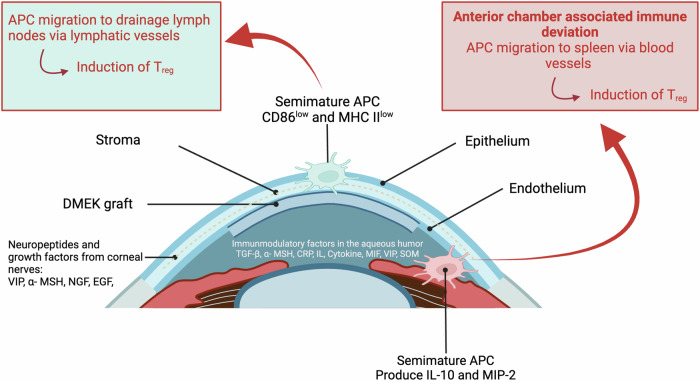
Schematic overview of Descemet Membrane Endothelial Keratoplasty (DMEK) and the anterior chamber associated immune deviation (ACAID). Created by S. Zwingelberg with Biorender.com.

In lamellar keratoplasty procedures, such as Descemet membrane endothelial keratoplasty (DMEK), the risk of transplant rejection is reduced due to the absence of direct contact with the immune system, a phenomenon attributed to the corneal privilege of avascularity. However, acute or chronic infections can still trigger rejection reaction (compare Fig. 3 and Fig. 4).

Understanding these mechanisms is crucial for developing strategies to mitigate transplant rejection and improve outcomes in corneal transplantation. Continued research in this area will contribute to advancements in transplant immunology and clinical practice.

### DMEK: Clinical procedure

**Fig. 5:**
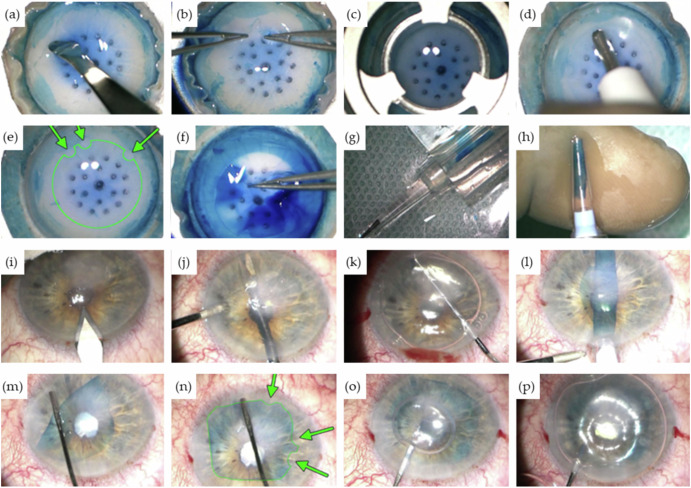
Step-by-step procedure of DMEK. **a** Circular rhexis of the peripheral Descemet membrane and staining with trypan blue followed by lifting of the Descemet edge. **b** Separation of the Descemet membrane using two forceps. **c** Trephination with an 8-mm trephine. **d**, **e** Placement of graft markings for intraoperative orientation using a 1 mm trephine (green arrows). **f** Peeling off the graft. **g**, **h** Loading the graft into the injector cartridge. **i** Making incisions. **j** Spreading the iridotomy with vitreous scissors. **k** Descemetorhexis. **l** Insertion of the graft. **m** Unfurling of the graft. **n** Verification of graft markings (green arrows). **o** Injection of the air bubble. **p** Filling with 20% sulfur hexafluoride (SF6) gas. (With the kind using permission of Prof. Dr. med. Claus Cursiefen and Prof. Dr. med. Björn Bachmann, University Hospital of Cologne, Department of Ophthalmology, Cologne, Germany).

By replacing only the dysfunctional endothelial layer, DMEK offers an effective, minimally invasive solution for corneal endothelial disorders (compare Figs. 4 and 5)^46–50^. Consequently, it has rapidly gained acceptance as the preferred surgical approach for treating endothelial dysfunction, with expanding indications beyond Fuchs endothelial corneal dystrophy (FECD) to include conditions such as pseudophakic bullous keratopathy, corneal decompensation, and failed previous grafts.

The DMEK procedure represents a significant advancement in corneal transplantation techniques, offering several advantages over traditional methods, including faster visual recovery, lower rates of rejection, and preservation of the corneal structure.

DMEK is a complex clinical procedure that requires a precise sequence of surgical steps to ensure successful outcomes. The detailed steps of the procedure are illustrated in Fig. 5 (a-p).

### DMEK: Advantages concerning the clinical outcome

Throughout clinical practice, the DMEK procedure has brought forth numerous advantages, which can be summarized as follows:

- Better Visual Acuity: DMEK consistently yields superior visual outcomes, often surpassing those achieved with previous techniques like Descemet’s Stripping Endothelial Keratoplasty (DSEK) and Penetrating Keratoplasty (PK). Patients benefit from improved visual clarity, contrast sensitivity, and reduced glare^48,55^.
- Faster Postoperative Recovery: Compared to traditional approaches, DMEK offers significantly shorter recovery times and quicker visual rehabilitation, allowing patients to resume daily activities more rapidly^48,49^.
- Lower Risk of Rejection: The targeted nature of DMEK, replacing only the dysfunctional endothelial layer, minimizes the antigenic exposure, leading to a reduced risk of immune-mediated rejection and better long-term graft survival^47^.
- Reduced Astigmatism: By preserving the recipient’s corneal structure, DMEK minimizes induced astigmatism, resulting in better refractive outcomes and less reliance on corrective lenses^48,56^.
- Improved Graft Stability: The ultra-thin DMEK grafts rapidly adhere to the recipient’s cornea, promoting stable integration and long-term durability^57–59^.

**Fig. 6:**
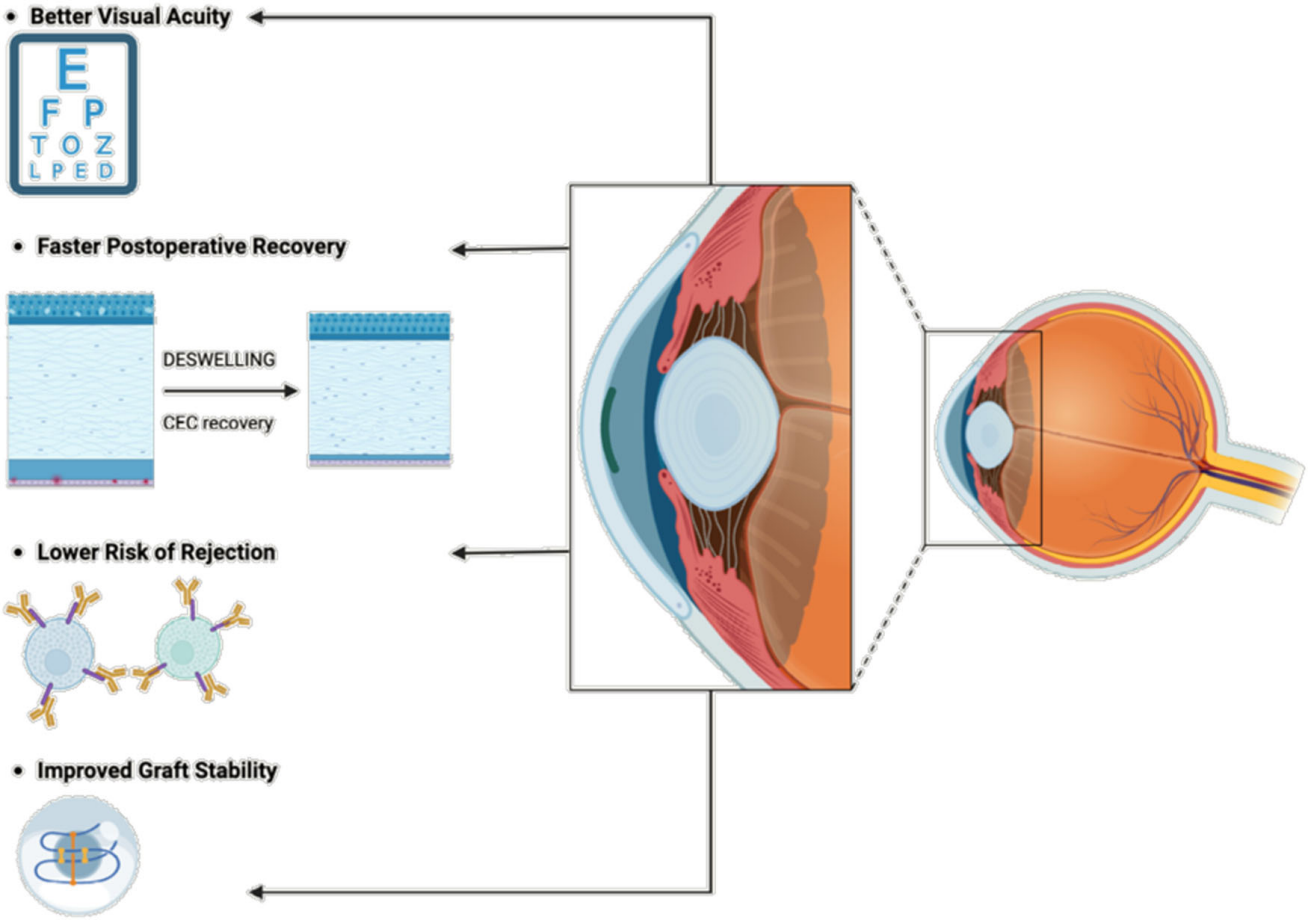
Schematic overview of the advantages of Descemet Membrane Endothelial Keratoplasty (DMEK). Created by S. Zwingelberg with Biorender.com.

Despite these significant advantages, DMEK poses some challenges (Fig. 6). Graft preparation and handling require exceptional surgical skill, and there are concerns regarding the availability of donor tissue^58,59^. However, advancements in surgical techniques, instrumentation, and postoperative care are continuously improving DMEK outcomes^58–60^. Additionally, innovations in tissue engineering and regenerative medicine may offer bioengineered corneal substitutes, potentially alleviating the shortage of donor tissue.

### DMEK: Requirements for grafts

**Fig. 7:**
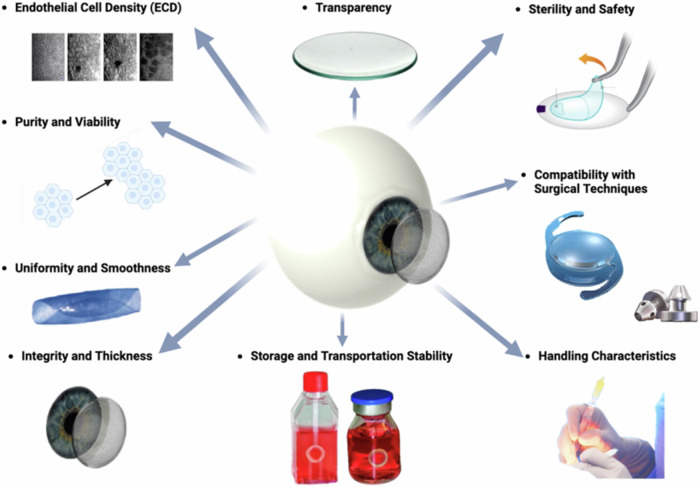
Schematic overview of the requirements for Descemet Membrane Endothelial Keratoplasty (DMEK). Created by S. Zwingelberg with Biorender.com.

Nevertheless, DMEK grafts remain essential components in the surgical treatment of corneal endothelial disorders (compare Fig. 7), though they may not be suitable for all situation^61^. These grafts must meet several stringent criteria to ensure successful transplantation and optimal visual outcomes for patients (compare Figs. 6 and 7)^62^:

- Integrity and Thickness: The Descemet membrane (DM) portion of the graft should be intact and free from tears, perforations, or damage. Additionally, the DM thickness should be within a specific range (approximately 15 μm) to maintain structural integrity while allowing for optimal endothelial cell function (15 μm)^63,64^.
- Endothelial Cell Density (ECD): The DMEK graft should contain a sufficient number of healthy endothelial cells to support corneal clarity and function post-transplantation (2000–3500 cells/mm³). Higher endothelial cell densities are associated with better graft survival and visual outcomes^65,66^.
- Transparency: Unlike most other tissues in the body, the cornea is avascular, meaning it lacks blood vessels. It instead receives oxygen and nutrients from the tear film on its outer surface and from the aqueous humor within the anterior chamber of the eye. This avascular nature helps reduce light absorption and maintain corneal transparency. The absence of pigmentation further enhances transparency by minimizing light absorption and scattering^67,68^. The corneal surface’s smoothness allows light to pass through with minimal distortion, critical for optimal visual clarity. Even minor irregularities or imperfections in the corneal surface can disrupt light passage, affecting visual acuity.
  The tear film covering the cornea helps maintain smoothness and optical clarity by providing a smooth refractive surface. Any disruption or opacification of the cornea—whether from injury, disease, or surgery—can lead to visual impairment and a diminished quality of life.
- Purity and Viability: The graft should be free from contaminants, debris, or cellular remnants that could provoke an immune response or impair endothelial cell function. The viability of endothelial cells within the graft is essential for their survival and proper function after transplantation^47^.
- Uniformity and Smoothness: The DMEK graft should exhibit uniform thickness and smoothness to facilitate handling and positioning during surgery. Irregularities or unevenness in the graft can cause difficulties in unfolding and adherence to the recipient’s cornea^69,70^. Factors such as stripping, splitting, rolling behavior, and fragility must also be considered^58,59^.
- Sterility and Safety: The sterility of the graft is paramount to preventing infection or disease transmission. Donor tissue must undergo thorough screening and processing to ensure safety and minimize the risk of adverse events post-transplantation.
- Handling Characteristics: DMEK grafts should have appropriate handling characteristics, allowing for ease of manipulation and placement in the recipient’s eye. Grafts that are too fragile or delicate may be prone to damage during surgical manipulation^58–60^.
- Storage and Transportation Stability: The grafts must maintain stability and viability during storage and transportation from the donor to the surgical site. Proper storage conditions, including controlled temperature and hydration levels, are essential to preserve graft quality.
- Compatibility with Surgical Techniques: The DMEK graft should be compatible with the surgical instruments and techniques used during transplantation. Grafts that are too rigid or fragile may pose challenges during surgical manipulation and insertion.

In summary, a successful DMEK graft must meet several key criteria, including structural integrity, endothelial cell density, purity, uniformity, sterility, handling characteristics, storage stability, and compatibility with surgical techniques. Adhering to these requirements ensures optimal outcomes for patients undergoing DMEK surgery, leading to improved visual acuity and quality of life.

### DMEK: Recent developments and future directions

**Fig. 8:**
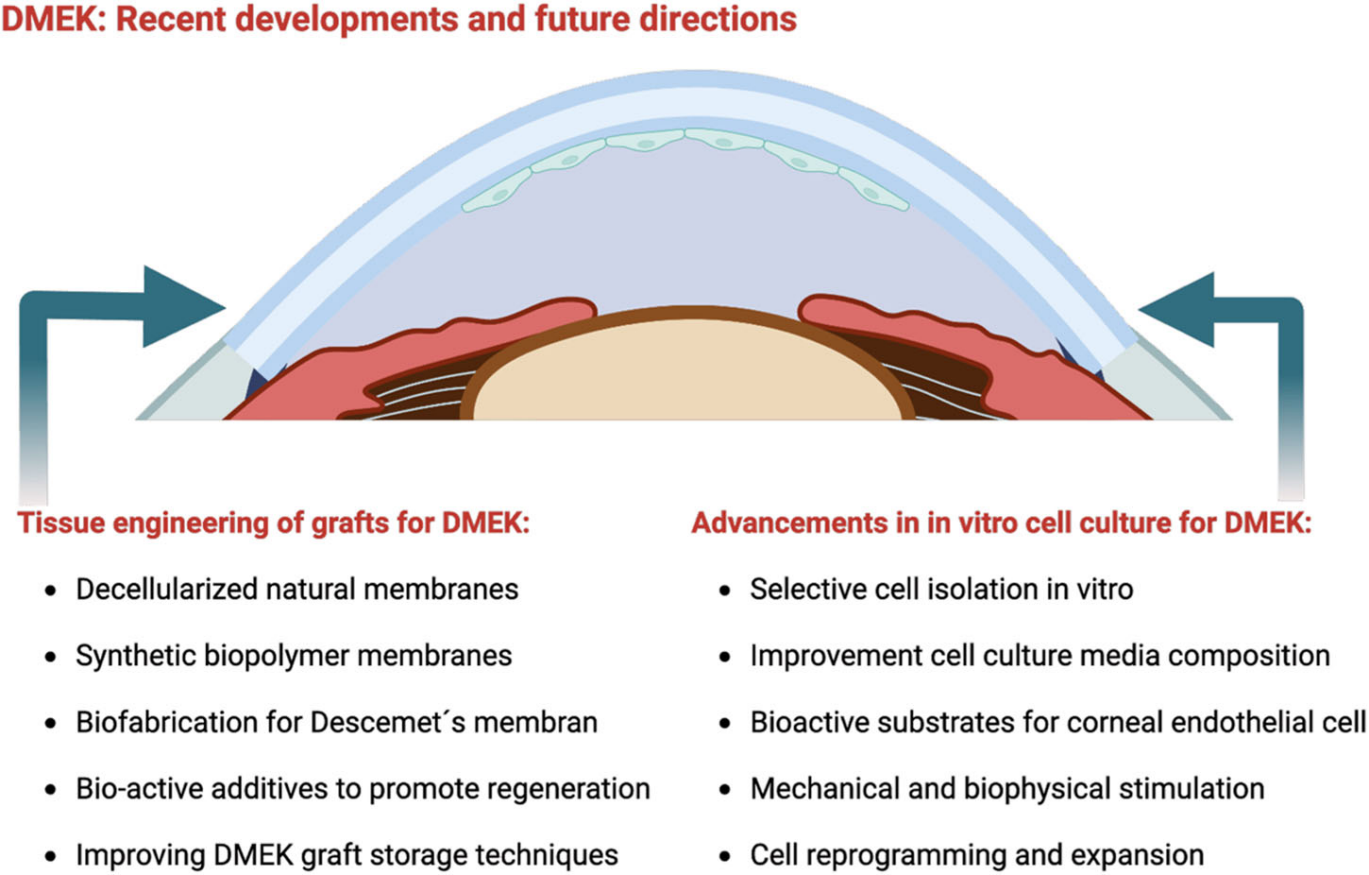
Schematic overview of recent developments and future directions for Descemet Membrane Endothelial Keratoplasty (DMEK). Created by S. Zwingelberg with Biorender.com.

Future directions in DMEK research include exploring personalized medicine, such as using patient-specific grafts tailored to individual patients, which could reduce the risk of rejection and improve outcomes. There is also significant interest in the integration of advanced imaging technologies to ensure precise graft sizing and positioning, increasing the accuracy of the procedure. Additionally, researchers are investigating the development of adjunctive therapies designed to enhance endothelial cell survival and maintain function post-transplant. Ongoing efforts aim to refine DMEK techniques further and tackle existing challenges (compare Fig. 8). This includes improving donor tissue preparation methods, advancing cell culture and bioengineering approaches, and exploring therapies that could boost graft survival and endothelial function in the long term.

### Advancements in in vitro cell culture for DMEK

The development of new cell culture methods for corneal endothelium (CE) has become an emerging research field aimed at overcoming the limitations of traditional corneal transplants and addressing the shortage of corneal donors^70^.

### Selective cell isolation for cell culture in vitro

**Fig. 9:**
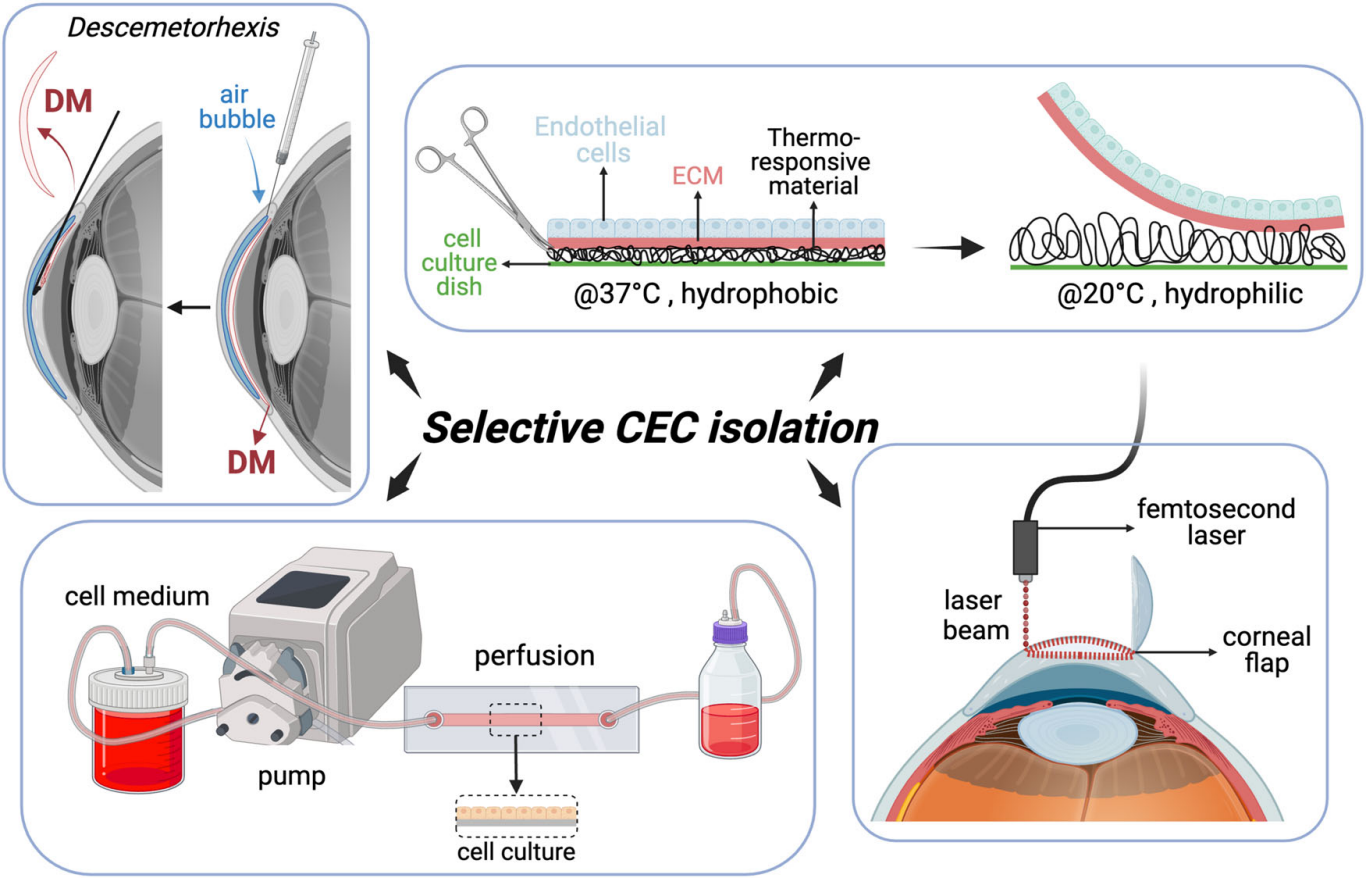
Schematic overview of Selective cell isolation for cell culture in vitro. Created by S. Zwingelberg with Biorender.com.

DMEK has emerged as a highly effective treatment for corneal endothelial dysfunction. However, the success of DMEK relies on the availability of healthy and functional corneal endothelial cells (CECs). One of the key challenges in DMEK surgery is obtaining a sufficient number of viable CECs for transplantation. In recent years, significant progress has been made in developing improved cell culture techniques to expand CEC populations ex vivo, providing a promising solution to address this challenge. Traditionally, isolating CECs has been challenging due to their delicate nature and the need to maintain cellular integrity. Techniques such as mechanical stripping or enzymatic digestion of Descemet’s membrane (DM) have been used but can lead to reduced cell viability and damage to the endothelial layer. Advanced techniques for selective cell isolation include (compare Fig. 9)^71–79^:

- Descemetorhexis with bubble technique (DEBUT): This novel method involves creating a small air bubble under the DM to detach it from the stroma. DEBUT allows for precise and controlled removal of the DM with attached endothelial cells, minimizing trauma to the tissue.
- Endothelial cell sheet harvesting: This approach involves harvesting CECs as intact cell sheets using specially designed spatulas or forceps. This method preserves cell-cell junctions and reduces cellular damage, leading to higher cell viability post-harvest.
- Temperature-responsive materials: Materials such as poly(N-isopropylacrylamide) (PIPAAm) simplify cell sheet preparation and transplantation by changing surface properties based on temperature.
  At 37 °C, PIPAAm is hydrophobic, promoting cell adhesion. When cooled below 32 °C, it becomes hydrophilic, allowing intact cell sheets to be harvested without damaging the extracellular matrix (ECM). While this method preserves cell viability, maintaining cells at 20 °C for extended periods can affect their function, prompting research into faster detachment methods.
- Femtosecond Laser-Assisted Techniques: Femtosecond lasers can create precise cuts along the DM, facilitating selective removal of the endothelial layer. This technique offers high precision and minimizes collateral damage to adjacent tissues.
- Perfusion and microfluidic devices: These systems have been developed to isolate CECs based on unique properties such as cell size or adhesion characteristics. They allow for automated and gentle cell isolation, improving the yield and purity of harvested cells.

Selective cell isolation techniques minimize trauma and maximize cell viability, ensuring the availability of healthy and functional CECs for transplantation. Moreover, higher-quality CECs obtained through selective isolation contribute to better visual outcomes and reduced risk of postoperative complications.

### Cell culture media composition

CECs have limited proliferative capacity in vivo, making it challenging to obtain a sufficient number of cells for transplantation. Moreover, maintaining the characteristic hexagonal morphology and pump function of CECs during ex vivo expansion is essential for successful transplantation outcomes. One of the major issues with primary isolated corneal endothelial cells using the techniques mentioned above is that CECs poorly proliferate in vivo, making it difficult to culture them in voter over the long term. Additionally, maintaining the proper phenotype and preventing endothelial-to-mesenchymal transition during culture expansion is problematic. CECs tend to lose their characteristic morphology and markers after just a few passages in proliferative media^80–82^. Furthermore, the global shortage of cornea donors and the fact that many factors, such as age, impact protocol standardizations necessitate the development of efficient culture methods to expand limited starting cells^82^. Therefore, multiple approaches are being explored to overcome the challenges of CEC culture, with the optimization of cell culture media being the most straightforward. Advanced cell culture media formulations containing growth factors, cytokines, and extracellular matrix components have been designed to support CEC proliferation while preserving cellular morphology and function. Optimized culture media typically contain the following components^83–86^:

- Growth factors: Growth factors such as basic fibroblast growth factor (bFGF), epidermal growth factor (EGF), and transforming growth factor-beta (TGF-β) play key roles in promoting CEC proliferation and maintaining cell viability. These factors stimulate cell division and support the growth of healthy CEC populations.
- Serum supplements: Fetal bovine serum (FBS) or human serum albumin (HSA) are commonly used as supplements in culture media to provide essential nutrients, hormones, and growth factors necessary for CEC growth and survival.
- Extracellular Matrix (ECM) components: ECM proteins like collagen, laminin, and fibronectin can be incorporated into culture media to mimic the natural environment of CECs and promote cell adhesion, migration, and differentiation.
- Osmolarity and pH control: Maintaining optimal osmolarity and pH levels in culture media is crucial for CEC health and function. Isotonic solutions and buffering agents are used to regulate osmotic pressure and maintain physiological pH.
- Antibiotics and antimycotics: The addition of antibiotics (e.g., penicillin-streptomycin) and antimycotics (e.g., amphotericin B) helps prevent microbial contamination and ensures the sterility of culture media.

In addition, advanced culture techniques are emerging as key future directions for selective cell isolation. Perfusion-based culture systems may allow for a continuous supply of nutrients and waste removal, improving cell growth kinetics and maintaining cellular homeostasis. Moreover, three-dimensional (3D) culture systems could better mimic the in vivo microenvironment of CECs, promoting cell-cell interactions and enhancing cell survival and function. Recent studies have explored the use of xenogeneic-free culture media as an effective method for culturing functional corneal endothelial cells^86^: T To bypass the original problem of isolating CECs and the low number of usable cells, various approaches for differentiating viable CEC-like cells from other, more readily available cells, such as embryonic stem cells, pluripotent stem cells, and even mesenchymal stem cells, are being investigated^71,87–89^:

### Bioactive substrates for CEC in vitro culture

CEC expansion for DMEK relies on innovative techniques to enhance cell adhesion, growth, and function in culture. One important strategy involves cell coating and substrate engineering, which optimize the culture environment to support CEC proliferation while preserving cellular phenotype and function. The success of CEC expansion in vitro is contingent upon replicating the physiological microenvironment of endothelial cells within the eye. Cell coating and substrate engineering aim to mimic this environment by providing structural and biochemical cues that promote cell adhesion, proliferation, and differentiation. Several key techniques in cell coating and substrate engineering include:

- Extracellular Matrix (ECM) coatings: ECM proteins such as collagen, fibronectin, laminin, and vitronectin are commonly used to coat culture surfaces. These proteins facilitate cell adhesion by interacting with specific integrin receptors on CECs, promoting cell spreading and survival^90–98^.
- Synthetic polymers: Synthetic polymers like polyethylene glycol (PEG) and poly(lactic-co-glycolic acid) (PLGA) can be modified to mimic ECM properties and enhance cell-substrate interactions. These materials offer tunable physical and chemical properties for tailored cell culture applications^91–95^.
- Nano-topography and surface modification: Surface roughness and topographical features can be engineered at the nanoscale to guide cell behavior. Nanostructured surfaces promote cell adhesion, alignment, and differentiation by influencing cytoskeletal organization and intracellular signaling pathway^93^.
- Biocompatible hydrogels: Hydrogel matrices provide a 3D scaffold for cell culture and can be functionalized with bioactive molecules to support CEC expansion. Hydrogels mimic the hydrated environment of the cornea and can sustain long-term cell viability and function^96–98^.

Optimized coatings and engineered substrates promote robust cell adhesion, minimizing cell detachment and loss during culture. Improved cell functionality can be achieved by mimicking the native ECM environment, which enhances CEC phenotype and function, preserving critical pump function and barrier integrity. Standardized coating protocols and engineered substrates facilitate large-scale CEC expansion for clinical applications, ensuring reproducibility and quality control.

### Mechanical and biophysical stimulation for CEC in vitro culture

The application of mechanical forces or biophysical cues, such as shear stress and substrate stiffness, has demonstrated significant potential in promoting CEC proliferation and maintaining cellular phenotype. CEC expansion is a crucial aspect of DMEK, necessitating innovative approaches to enhance cell proliferation and preserve cellular function in vitro. Mechanical and biophysical stimuli represent promising strategies to modulate CEC behavior and facilitate cell expansion for transplantation^87,99–104^. Given that the corneal endothelium is constantly subjected to mechanical forces within the eye, which influence cellular behavior and function, replicating these physiological cues in culture can enhance CEC growth, morphology, and phenotype, ultimately improving transplant success. Key techniques in mechanical and biophysical stimulation include:

- Fluid shear stress: Mimicking aqueous humor flow in the eye, fluid shear stress can be applied to CECs using perfusion bioreactors. This mechanical stimulation promotes cell alignment, elongation, and proliferation by activating mechanosensitive pathways.
- Substrate stiffness: The stiffness of the culture substrate can influence CEC behavior. Soft substrates that resemble the elasticity of native corneal tissue promote cell spreading and help maintain the endothelial phenotype, while stiffer substrates can induce cell differentiation.
- Mechanical stretch: Controlled mechanical stretching of cell monolayers can enhance CEC proliferation and extracellular matrix production. Stretch-induced mechanotransduction pathways regulate gene expression and cellular responses.
- Micropatterning and topographical cues: Nanoscale or microscale surface patterns can guide CEC alignment and morphology. Substrate micropatterning influences cytoskeletal organization and focal adhesion dynamics, impacting cell adhesion and function.

Mechanical stimulation encourages uniform CEC alignment, resembling the native tissue architecture critical for corneal transparency. Biophysical cues support the maintenance of endothelial barrier integrity, preserving pump function and preventing corneal edema. Biomechanical cues stimulate CEC proliferation and metabolic activity, facilitating the generation of transplantable cell populations. Further exploration of mechanical and biophysical stimuli in CEC culture aims to optimize conditions for DMEK applications. Integrating these techniques into tissue engineering strategies holds promise for enhancing CEC expansion efficiency, improving transplant outcomes, and advancing regenerative therapies for corneal endothelial dysfunction.

### Cell reprogramming and expansion in cell culture in vitro

Innovative strategies utilizing cell reprogramming techniques, such as induced pluripotent stem cells (iPSCs) and cell transdifferentiation, have been explored to generate functional CECs in large quantities. CEC expansion is a critical aspect of DMEK, and recent advancements in cell reprogramming techniques offer promising approaches to produce sufficient CECs for transplantation. Possible cell reprogramming strategies include:

- Induced Pluripotent Stem Cells (iPSCs): iPSC technology involves reprogramming somatic cells, such as skin fibroblasts or peripheral blood cells, into a pluripotent state using defined factors (e.g., Oct4, Sox2, Klf4, c-Myc). These iPSCs can then be differentiated into CEC-like cells through stepwise induction protocols^105–107^.
- Direct cell conversion (Transdifferentiation): Transdifferentiation strategies aim to directly convert one cell type into another without passing through a pluripotent state. For example, fibroblasts or other cell types can be directly reprogrammed into CEC-like cells using specific transcription factors or small molecules^108–111^.
- Gene editing and correction: Genome editing technologies like CRISPR/Cas9 can be used to correct genetic mutations associated with CEC dysfunction, enabling the generation of healthy CEC populations suitable for transplantation^112,113^.

Optimization of expansion protocols should focus on defined culture conditions, quality control, and characterization

- Culturing reprogrammed or transdifferentiated CECs in specialized media containing growth factors, hormones, and extracellular matrix components that mimic the native corneal microenvironment.
- Comprehensive characterization of expanded CECs to ensure the preservation of endothelial phenotype, morphology, and functional properties essential for corneal transparency and pump function.

The clinical implications and benefits include the potential of cell reprogramming techniques to generate patient-specific CECs, thereby reducing the risk of immune rejection and broadening the donor pool for DMEK procedures. Scalable expansion protocols further enable the production of ample quantities of functional CECs, meeting the demand for transplantable cells in corneal regenerative medicine.

### Tissue engineering of grafts for DMEK

Cell injection therapy and cell sheet technology are two scaffold-free approaches for regenerating healthy corneal endothelium. In cell injection therapy, cultured CECs are directly injected into the posterior corneal surface without any carrier scaffold. The injection of human CECs supplemented with a Rho-associated kinase (ROCK) inhibitor (discussed later) was implemented in bullous keratopathy patients, resulting in increased cell density after 24 weeks in 11 patients. Although clinical trials of human CEC injection have shown promising results, safety issues remain a concern. Since the cells are not bound to any scaffold, the fate of unattached CECs after injection into the cornea is not fully understood. It is speculated that unattached CECs may enter the systemic circulation via adjacent veins, potentially leading to tumor formation^70^. n cell sheet engineering, cell layers are formed by cultivating human corneal endothelial cells on a culture dish coated with stimuli-responsive polymers. Confluent cell sheets are then detached from the polymer surface using a stimulus. A major limitation of this method is that cell sheets have weak mechanical strength, making them prone to shrinkage or rupture during detachment, which challenges the preservation of cell–cell and cell–extracellular matrix (ECM) interactions as well as the integrity of the cell sheets^114^.

To circumvent these risks, extensive research has been conducted to develop mechanically stable and biofunctional membranes that meet the implant criteria for DMEK, as outlined in Chapter 2.3. The main idea driving scaffold-based strategies is to imitate the ECM of the native tissue using biomaterials to create a suitable microenvironment for effective cell regeneration^115^. Tissue engineering scaffolds function as templates and signaling supports, providing adhesion, growth, migration, and proliferation for cells. An ideal scaffold in tissue engineering should closely resemble natural tissue in terms of permeability, transparency, porosity, biocompatibility, and morphology^115,116^.

DM is formed by ECM components (such as collagen IV-VIII, fibronectin, and laminin) secreted by CECs, supporting the growth and function of corneal endothelial cells. Alterations in the composition or structure of DM are a primary cause of corneal endothelial diseases like Fuchs’ endothelial corneal dystrophy (FECD). Since DM is the native substrate for the optimal functionality of human endothelial cells and the regeneration of healthy corneal endothelium, replicating the true features of natural DM—biological, mechanical, and structural properties—is essential^117,118^.

For DMEK, the desired scaffold should be thin, transparent, permeable, and highly flexible to enable its insertion under the cornea by rolling it up^119^. The following sections discuss various types of grafts, both of natural origin and synthetic strategies, to meet the high requirements for DM grafts.

### Artificial membranes as grafts for DMEK

Corneal endothelial transplantation is essential for treating corneal diseases, and selecting appropriate graft materials plays a pivotal role in the procedure’s success. Various biomaterials are utilized for corneal scaffold engineering, including synthetic biopolymers such as polycaprolactone (PCL), polyethylene glycol (PEG), and polyvinyl alcohol (PVA), as well as natural biomaterials like gelatin, chitosan, hyaluronic acid, silk, alginate, cellulose, and collagen^114–119^. Different cell types exhibit distinct preferences for these materials, highlighting the importance of matching scaffold materials to cell needs^116^. Effective attachment to the posterior corneal surface during healing is crucial for transplant success.

Hydrophilic scaffolds, such as chitosan, gelatin, collagen, and PEG, enhance attachment and reduce the need for additional binding agents^120^. Natural biopolymers like collagen and chitosan mimic the extracellular matrix, promoting biocompatibility while reducing inflammation and infection risks. Synthetic polymers such as polycaprolactone (PCL) and polyethylene glycol (PEG) can be tailored for mechanical strength but lack the natural signals necessary for immune tolerance. Combining synthetic and natural materials, often with embedded anti-inflammatory agents, enhances both integration and immune modulation, improving graft success in corneal regeneration

### Natural biomaterials

closely mimic the body’s own components, minimizing inflammatory reactions, especially when processed to be inert. collagen and glycosaminoglycans, for instance, offer excellent biocompatibility, biodegradability, and non-toxicity. Moreover, natural materials like chitosan also provide antimicrobial and anti-inflammatory properties, which prevent microbial growth and promote wound healing^119,121^. Despite chitosan’s poor mechanical properties, it can be modified by crosslinking or combined with other biopolymers^119^. Combining different materials is a common strategy to overcome the limitations of individual biomaterials^119,122^. Frequently, natural and synthetic materials are combined to achieve an optimal balance of mechanical strength, biocompatibility, and transparency.

### Synthetic polymers

offer better control over mechanical properties and transparency. One commercial example, EndoArt®, is made from a flexible acrylic material that forms an impermeable barrier to liquid transfer. EndoArt® fits the curvature of the cornea with a thickness of 50 μm and is attached to the posterior corneal surface using an air-bubble technique with C_3_F_8_. Early clinical results have demonstrated significant improvements in reducing edema, pain, and visual clarity. However, frequent detachment of the device has been reported, necessitating additional air-bubbling or suturing. Moreover, EndoArt’s lack of corneal endothelial cell (CEC) migration limits its effectiveness, especially in the peripheral cornea^123,124^.

An unresolved issue is whether EndoArt’s impermeability might impact long-term nutrient transfer, essential for maintaining corneal health^125^. While promising as an alternative to traditional endothelial grafts, it is currently reserved for severe or palliative cases, particularly in patients with multiple graft rejections. To improve its clinical applicability, more extensive studies involving larger patient populations and longer follow-up periods are necessary, alongside enhancements in adhesion and nutrient permeability^123,124^. In research, synthetic biopolymers like PCL, PEG, and PVA are often used to develop corneal scaffolds.

These materials allow for better control over mechanical strength compared to natural biomaterials. However, they typically lack essential cell-recognition signals necessary for cell proliferation, adhesion, and differentiation. This deficiency can lead to issues such as biofilm formation and fibrosis, which can result in infections and immune responses. PCL is widely used for its mechanical strength and, when combined with natural materials, can improve scaffold properties for corneal applications. PEG is another significant synthetic polymer, known for its hydrophilicity, which improves cell attachment to the cornea, reducing the need for stitches and additional binding agents. Similarly, PVA enhances interaction between cells and the corneal surface, contributing to the advancement of corneal tissue engineering.

### Blending natural and synthetic materials

can overcome the limitations inherent in each type, yielding superior properties for corneal scaffolds. One example involves chitosan-PCL blends, which incorporate chitosan nanoparticles for cultivating human corneal endothelial cells (CECs).

The addition of PCL enhances chitosan’s molecular properties, while chitosan nanoparticles improve surface characteristics and cellular attachment^116^. The transparency of the composite membrane improves as PCL content decreases, a crucial factor for corneal engineering. Human CECs isolated from Descemet’s membrane were cultured with a ROCK inhibitor to promote proliferation and adhesion. Results demonstrated that specific combinations of chitosan, PCL, and chitosan nanoparticles improved scaffold characteristics, enhancing adhesion, survival, and proliferation, while maintaining biocompatibility and biodegradability. In another approach, hydroxyethyl chitosan, modified for enhanced biocompatibility and solubility, was combined with gelatin and chondroitin sulfate to create CEC carriers for corneal tissue engineering^126^. This blend membrane demonstrated significant permeability, transparency, water content, and degradability. Rabbit CECs seeded onto the blend membrane showed good cytocompatibility, attachment, and proliferation, suggesting potential for rabbit CEC regeneration. However, further research is needed to assess its impact on human CECs. In a different study, Rafat et al. combined chitosan and collagen, crosslinked via EDC/NHS or PEG-dialdehyde/EDC/NHS, for corneal tissue engineering^127^. Thick hybrid membranes (∼500 μm) were tested in vitro with immortalized human corneal epithelial cells and in vivo on pig eyes, demonstrating desirable mechanical properties, optical clarity, suturability, and permeability. The inclusion of chitosan significantly improved toughness and elasticity, making the membrane suitable for corneal implantation. Future studies may focus on developing thinner membranes for use as endothelial cell carriers in DMEK. Similarly, Ozcelik et al. fabricated ultrathin chitosan-PEG hydrogel films (∼50 μm) for CEC regeneration and implantation^128^. These PEG-crosslinked chitosan polymers exhibited high optical transmission and permeability, demonstrating potential for ophthalmic tissue engineering. Sheep CECs cultured on these films showed excellent biodegradability, attachment, and proliferation. Ex vivo trials on sheep eyes confirmed the feasibility of CEC transplantation.

### Nanofibrous membranes

produced via electrospinning are an alternative strategy that results in porous grafts. One such approach used gelatin as the primary component for CEC transplantation^129^. Gelatin nanofibers were produced through electrospinning, followed by vapor crosslinking with glutaraldehyde.

These nanofiber membranes supported CEC culture and were used for DMEK in rabbit eyes. Both immortalized human corneal endothelial cells and primary rabbit corneal endothelial cells were employed in vitro. The findings demonstrated non-toxicity and functional protein expression, with successful proliferation and migration on the electrospun gelatin nanofibers. However, rapid degradation of the gelatin membrane could limit CEC attachment and migration.

Additionally, electrospun silk nanofibers hold promise for ocular regeneration due to their non-toxicity, transparency, biodegradability, biocompatibility, and permeability^130^. However, in vivo studies have shown that they may induce vascularization^131^. To mitigate this, silk has been combined with other polymers or bioactive molecules. For instance, Forouzideh et al. incorporated epigallocatechin gallate (EG) into silk fibroin to prevent angiogenesis during ocular regeneration^132^. Electrospinning was used to fabricate EG-silk nanofibers with a high surface-to-volume ratio and porosity. The study revealed that the electrospun scaffold promoted adhesion and proliferation of corneal limbal cells. Angiogenesis evaluation showed controlled EG release for 144 h and dose-dependent inhibition of human umbilical vein endothelial cells. These findings indicate that EG-silk nanofibers hold potential as scaffolds for endothelial tissue engineering and the delivery of anti-angiogenic materials.

### A-/ Decellularized natural membranes as grafts for DMEK

One option for utilizing grafts in DMEK involves donor material, such as allografts or porcine xenografts. The Descemetorhexis technique, which entails stripping a portion of Descemet’s membrane (DM), is believed to stimulate central endothelial cells to migrate and form a monolayer, thereby restoring endothelial function. Additionally, the transplantation of a healthy basement membrane onto the stripped area may further accelerate the healing process. Bhogal et al. demonstrated that transplanting decellularized DM onto the stripped area supports corneal endothelial cell (CEC) reproduction and migration by serving as a scaffold. This approach effectively reduced edema, restored normal corneal thickness and clarity, and accelerated wound healing compared to cases without the acellular DM graft^123,133^.

The porcine cornea is frequently used in tissue engineering due to its biological and functional similarities to the human cornea, as well as its availability as an unlimited source^134^. Various studies have employed decellularized DM^135,136^ or porcine corneal stroma^17,134,137^ as substrates for endothelial keratoplasty. These tissues are meticulously separated from epithelial and endothelial cells and decellularized using several methods. For instance, one study used 100% human serum followed by electrophoresis to decellularize porcine stroma, which prevented corneal denaturation and opacification. In vitro tests demonstrated no cytotoxic effects on human corneal epithelial cells, and short-term in vivo results in mice were successful, with no cases of rejection. The acellular stromal membrane obtained through this decellularization technique may present a promising option for corneal endothelial tissue engineering^137^.

The most common natural membranes used as scaffolds in the biofabrication of corneal endothelium include placental amniotic membrane, corneal DM, and other decellularized corneal membranes^114,119^. Therefore, it is crucial that the required decellularization process does not cause functionally impairing damage to the donor membrane.

The physicochemical properties of DM are essential for its biofunctionality, and characteristics such as biomechanics must be largely preserved during decellularization. To achieve this, the following methodological aspects must be considered^138–142^:

- Decellularization agents: A combination of detergents (e.g., Triton X-100, sodium dodecyl sulfate) and enzymatic treatments (e.g., trypsin, DNase) is used to remove cellular components from DM while minimizing damage to the extracellular matrix (ECM).
- Perfusion and dynamic systems: Perfusing decellularization agents through the DM using dynamic systems (e.g., bioreactors, flow chambers) enhances agent penetration and distribution, improving the efficiency and uniformity of decellularization.
- Optimization of parameters: Parameters such as temperature, pH, and duration of the decellularization process must be optimized to achieve maximal cell removal while preserving ECM structure and biomechanical properties.
- Biophysical methods: Mechanical agitation or ultrasound-assisted techniques can aid in cell removal and enhance decellularization efficiency without compromising the integrity of DM.

The evaluation of biomechanical properties, such as tensile strength analysis, must be conducted to assess the resistance of decellularized DM to deformation and rupture, indicating the preservation of structural integrity. Additionally, spectrophotometric analysis can evaluate the transparency and light transmission characteristics of decellularized DM, which are crucial for visual function and clinical outcomes in DMEK. Decellularized DM that retains its biomechanical properties exhibits reduced immunogenicity, promoting biocompatibility and minimizing the host immune response following transplantation. The retention of biomechanical properties also facilitates the surgical handling and manipulation of decellularized DM grafts during DMEK procedures, thereby enhancing surgical outcomes and patient recovery. Continued research into enhanced decellularization protocols may help optimize the preservation of biomechanical properties in DM for corneal endothelial transplantation. Integrating advanced techniques and comprehensive characterization methods can drive the development of next-generation tissue-engineered constructs, advancing the field of regenerative medicine in ophthalmology. However, significant limitations remain, such as a shortage of donors, low quality of donor tissue, uncertain and variable membrane compositions, contamination or infection risks, and the potential disruption of tissue integrity during sterilization, decellularization, and preservation^139,143^. Moreover, important signaling molecules essential for corneal health (such as growth factors, cytokines, and proteins) are likely denatured or eliminated during the decellularization process of natural membranes. A reliable and standardized protocol for the decellularization of human corneas has yet to be established. Consequently, achieving complete removal of cellular materials while maintaining membrane integrity presents a significant challenge^143^.

### Bio-active additives to promote DM regeneration

The human corneal endothelium (CE) has a limited regenerative capacity due to decreased mitotic activity with age. Therefore, stimulating the proliferation of corneal endothelial cells (CECs) using signaling molecules is essential for effective regeneration in cases of CE defects.

Signaling molecules—including growth factors (GFs), cytokines, small chemical compounds, and coding mRNAs—play critical roles in cell adhesion, differentiation, migration, and function while also preventing inflammation by reactivating cellular signaling pathways involved in cell-cell and cell-scaffold interactions. These molecules are frequently used as therapeutic agents, either alone or in combination with cells or scaffolds.

CE tissue engineering scaffolds are typically composed of water-soluble materials that can encapsulate signaling molecules or facilitate their binding. Ideally, these scaffolds should provide continuous release of signaling molecules to cells after implantation^120^:

- Growth factors to trigger cellular pathways between CECs: Growth factors operate by binding to specific high-affinity receptors on cell surfaces to activate signaling pathways. The combination, concentration, dose, formulation, and release timing of these signaling molecules are crucial for successful CE healing. Various studies have shown that fibroblast growth factor (FGF), basic fibroblast growth factor (bFGF or FGF2), epidermal growth factor (EGF), hepatocyte growth factor (HGF), and nerve growth factor (NGF) can promote the proliferation of CECs directly or indirectly. Some GFs, such as insulin-like growth factor (IGF-1), can enhance the effects of others, notably contributing to EGF’s role in CEC proliferation^132,144,145^.
- Cytokines to prevent inflammation in CE: Previous research has indicated that some DMEK cases can trigger immune responses in the host cornea following transplantation, which may ultimately lead to graft failure. While there is limited research on specific inflammatory and anti-inflammatory cytokines involved in CE defects, immune responses have been linked to increased concentrations of certain inflammatory cytokines (such as IL-8) and the recruitment of macrophages (like MCP-3) after DMEK surgery. To inhibit these inflammatory cytokines and prevent inflammation in defective CE, anti-inflammatory cytokines (e.g., IL-4 and IL-13) can be incorporated into biofabricated scaffolds as signaling molecule^146,147^.
- Other anti-inflammatory or bioactive compounds to enhance wound healing: Beyond anti-inflammatory cytokines, various anti-inflammatory eye drops containing mydriatics, nonsteroidal, and steroidal compounds have been used to treat CE. Their protective effects, such as reducing inflammation and increasing CEC activity, have been observed with frequent use. However, eye drops are not an ideal delivery system for ocular drugs due to the need for prolonged and frequent application, coupled with significant drug loss during administration. Particularly for corneal endothelial diseases, eye drops are often ineffective because the active compounds do not adequately reach the posterior part of the eye. To reduce reliance on eye drops, these compounds can be incorporated into or onto biofabricated scaffold structures to enable controlled drug release over time^148,149^

Another compound commonly used in eye drops to accelerate endothelial wound healing is the Rho-associated kinase (ROCK) inhibitor. In addition to the clinical trials of CEC injection therapy that utilize ROCK inhibitors, findings by Okumura and colleagues have shown that ROCK inhibitors can promote the regeneration of a corneal endothelial monolayer with an increased CEC density by enhancing cell adhesion and proliferation while suppressing CEC apoptosis, both in vitro and in vivo^150,151^. In addition to their application in eye drops, ROCK inhibitors can also be incorporated into suitable scaffolds to support the effective biofabrication of CE.

### Biofabrication in the context of DM grafting

**Fig. 10:**
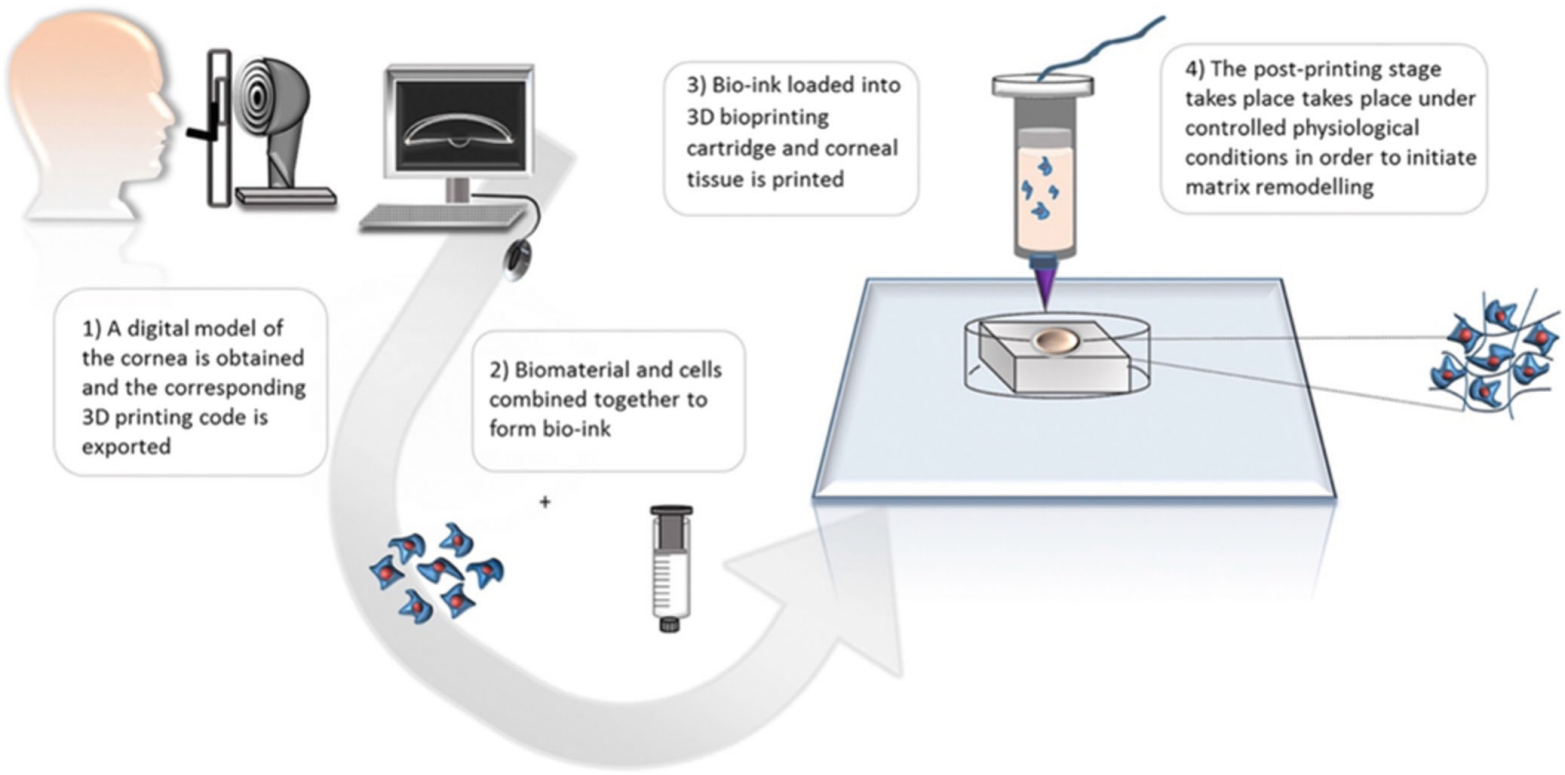
Model of 3D-bioprinting for engineering of artificial corneal structures (From: Isaacson A, Swioklo S, Connon CJ. 3D bioprinting of a corneal stroma equivalent^153^.

3D printing technology offers the capability to produce biomaterials with high resolution, allowing for the creation of patient-specific products with diverse patterns generated layer by layer using computer-aided design (CAD) digital models (see Fig. 10). Although many studies have explored 3D bioprinting for the regeneration of epithelium and stroma, its application in corneal endothelial (CE) regeneration remains relatively under-researched. The three-dimensional structure, thickness, shape, topology, and mechanical properties of Descemet’s membrane (DM) are critical parameters that directly influence the activity, attachment, proliferation, differentiation, and migration of corneal endothelial cells (CECs). Additionally, both the corneal endothelium and DM are thin and avascular layers, which makes 3D bioprinting particularly promising for producing scaffolds that closely mimic the natural DM. One of the significant challenges in this field is identifying the right biomaterials that closely resemble native tissue characteristics suitable for various 3D printing techniques. Different biomaterials can be utilized across a range of 3D printing methods, making material selection a key consideration in achieving successful outcomes in CE regeneration^152,153^.

Due to the non-proliferative nature of corneal endothelial cells (CECs), only two studies focusing on the 3D bioprinting of CECs have been published to date. The first attempt at 3D bioprinting CECs occurred in 2018, utilizing extrusion-based 3D printing techniques. In this study, human CECs, transfected with an RNase 5 vector, were cultured and suspended in a gelatin-based bioink. The cells were then printed onto decellularized amniotic membranes, achieving a dense and homogeneous distribution. The results indicated improved proliferation and functionality of the 3D bioprinted constructs^154^.

A more recent study published on March 14, 2024, involved bioprinting CECs differentiated from human pluripotent stem cells using a covalently crosslinked hyaluronic acid bioink via an extrusion-based 3D printer. This study evaluated shape fidelity, printability, biocompatibility, and bioink integration. The findings confirmed biocompatibility, demonstrated high integration of the bioink with the cells on human Descemet’s membrane, and showed improvements in cell viability, morphology, and function^155^.

As 3D printing for corneal endothelium regeneration is still a nascent field, there have been no clinical trials to date, to the best of our knowledge. To effectively create and implement an artificial corneal endothelium in clinical settings, it is essential to consider the clinical needs for Descemet’s Membrane Endothelial Keratoplasty (DMEK). Therefore, collaboration between scientists and ophthalmologists is crucial for the successful translation of artificial scaffolds from the laboratory to the clinic.

### Improving DMEK graft quality through dynamic storage techniques

In addition to in vitro cell culture approaches, bioreactors present a promising option for enhancing corneal graft quality, thus expanding the pool of grafts suitable for DMEK. In vivo, CECs are subjected to mechanical forces, such as intraocular pressure (IOP) and aqueous humor flow, which induce shear stress^156^.

Conventional static storage techniques include long-term organ culture (OC) at 31–37 °C and short-term hypothermic storage (HS) at 2–6 °C^157^. In both methods, the cornea is isolated from the eyeball and immersed in the respective storage medium. However, these passive storage techniques fail to accurately replicate the physiological forces experienced by CECs in vivo, resulting in significant endothelial cell loss during storage and limited graft availability for DMEK^158^. Dynamic storage of corneal transplants can address these issues through two primary approaches:

- Application of IOP: In dynamic cultivation systems, pressure sensors enable precise regulation of peristaltic pump speeds, allowing for the maintenance of physiological IOP levels, typically ranging from 7 to 21.5 mmHg^159,160^.
- Continuous medium flow: Traditional media used during OC and HS, such as Eagle’s Minimum Essential Medium (MEM) with 2–8% fetal bovine serum (FBS) and Optisol-GS, do not provide consistent exposure to fresh medium. In OC, medium changes occur infrequently, often not at all, or only every 1-2 weeks^161^. Dynamic cultivation in flow bioreactors ensures continuous medium renewal, enhancing nutrient transport and metabolic waste removal compared to static storage methods. Additionally, separate media circuits can facilitate the use of epithelium- and endothelium-specific media tailored to enhance CEC viability, including serum-free media^160,162^.

These dynamic conditions have been shown to significantly improve CEC survival and function, as evidenced by increased Na + /K+ ATPase expression and enhanced cell morphology^160^. In conclusion, the controlled dynamic environment provided by bioreactors, characterized by improved nutrient supply and mechanical stimulation, represents a promising platform for the cultivation and preservation of DMEK grafts from ex vivo corneal tissue. Furthermore, this platform has the potential to be utilized for the cultivation of tissue-engineered DMEK grafts, potentially improving their overall quality.

### Cellular therapies as an alternative strategy for DMEK procedure

In addition to traditional tissue engineering approaches, scaffold-free methods are being developed to restore corneal endothelial function. These innovative strategies aim to reduce complications and enhance the integration of therapeutic cells, and they hold promise for autologous cell therapies, especially in situations where donor tissue is unavailable or unsuitable.

- Anterior eye injection of cultured CECs*:* In cell injection therapy, cultured CECs are injected directly into the posterior corneal surface without the use of a carrier scaffold. In a study involving patients with bullous keratopathy, human CECs supplemented with a Rho-associated kinase (ROCK) inhibitor were injected, resulting in increased cell density after 24 weeks in 11 patients. ROCK inhibitors, such as Y-27632, help maintain CEC integrity by preventing the formation of stress fibers associated with epithelial-mesenchymal transition (EMT), which can compromise CEC integrity by disrupting cell polarity and intercellular junctions. Preventing EMT is crucial for preserving the functional characteristics of CECs. While these clinical trials have shown promising results, safety concerns persist, as the behavior of unattached CECs post-injection remains unclear. There is speculation that these unattached cells could enter the systemic circulation through adjacent veins, potentially leading to tumor formation^113^. To improve cell retention and integration, new magnetic guidance systems have been developed. In a rabbit model, superparamagnetic nanoparticles attached to CECs localized the cells to the intact Descemet’s membrane, minimizing migration and enhancing integration^163^. This approach could help address some challenges associated with unattached cells, thereby improving the efficacy and safety of CEC injection therapy.
- Induced Pluripotent Stem Cells for cell therapies: The use of iPSCs presents a promising avenue for developing autologous therapies^33^. While clinical trials have involved autologous cultivated epithelial cell sheets for patients with limbal stem cell deficiencies, comparable studies using CECs are still lacking. iPSCs can be differentiated into neural crest cells (NCCs) and subsequently into corneal endothelial cells (CECs). Successful differentiation has been confirmed by the expression of key markers associated with human CECs, such as Na + /K + -ATPase, ZO-1, AQP1, Vimentin, *COL4A1*, *COL8A1*, and *COL8A2*^35,46,47^. iPSC-derived corneal endothelial cell substitutes (CECSi) have demonstrated the ability to improve corneal transparency in a monkey corneal edema model, although some animals exhibited immune rejection responses^48^. Establishing reliable differentiation protocols for iPSC-derived CECs presents challenges, particularly in optimizing xeno-free media and achieving consistency across various formulations^49^. Standardizing the processes for the derivation and differentiation of iPSCs is crucial to ensure their safe and consistent application in corneal tissue engineering and scaffold-free methodologies, emphasizing the need to adhere to Good Manufacturing Practice (GMP) guidelines to ensure safety and efficacy in clinical applications.
- Gene therapy strategies for corneal endothelial function: Gene therapy presents another approach to enhancing CEC function. Both ex vivo and in vivo strategies are being investigated to improve graft quality and address endothelial dysfunction. In ex vivo gene therapy, the goal is to modify CECs directly on the Descemet’s membrane of donor corneas prior to transplantation. Viral vectors, including adenoviruses and lentiviruses, have been utilized to facilitate gene transfer to CECs^50,55^. Research has shown that transfecting human donor corneas with anti-apoptotic genes, such as *BCL-xL* or *p53*, enhances CEC survival and helps preserve DMEK grafts during cultivation^56^. Additionally, the transfection of oncogenic genes, like *E2F2*, has been found to transiently increase CEC density while maintaining the hexagonal shape and monolayer structure of the cells^57^. In vivo, the CRISPR/dCas9 system has emerged as a potential tool for promoting CEC function. By overexpressing *SOX2*, a key transcription factor associated with stemness and critical for iPSC reprogramming, researchers have observed increases in both the proliferation and survival of CECs^58^.

Overall, the functionality of maintaining corneal transparency through the barrier and pump functions of the corneal endothelium is fundamentally dependent on its structural integrity, specifically a monolayer of hexagonal CECs. Although human CECs show regenerative potential for in vitro expansion, their proliferative capacity is limited, and they are prone to undergo EMT. A recent study by Lu et al.^135^. simulated EMT by adding TGF-β1 to the culture medium of human CECs, aiming to identify differentially expressed genes (DEGs) through RNA sequencing. The findings enhance understanding of EMT mechanisms during CEC cultivation and highlight potential targets for improving in vitro expansion and maintaining phenotypic integrity. However, the complexity of these processes necessitates further studies, which should be considered in the broader context of the various aspects discussed in this review.

## Discussion

In recent years, Descemet Membrane Endothelial Keratoplasty (DMEK) has emerged as the new gold standard for treating corneal endothelial disorders. As healthcare continues to advance, improving quality and safety standards, the need for standardized techniques in regenerative medicine becomes increasingly apparent. The selection of materials for implants is crucial, with a strong focus on their origin and properties.

The functionality of the deendothelialized Descemet membrane (DM) relies on several critical factors, including transparency, thickness, biomechanics, and permeability. Corneal endothelial cells are responsible for synthesizing DM through the precise arrangement of components such as collagen IV-VIII, fibronectin, and laminin. In cases requiring DMEK, replacement membranes must closely mimic the properties of natural DM to enable endothelial cells to regain their natural function and reconstruct a functional DM. Traditional methods using donor-derived membranes have shown promising results; however, addressing the increasing demand for membranes amid a growing donor shortage and evolving safety standards poses a significant challenge. Endothelial transplantation, including DMEK, is indicated for various conditions beyond Fuchs’ endothelial corneal dystrophy (FECD), such as pseudophakic bullous keratopathy—which is currently under investigation for ROCK inhibitor therapy—as well as conditions with glaucomatous or inflammatory origins. This broad range of indications opens up extensive potential applications for artificial corneal endothelial replacement therapy.

Tissue engineering techniques that allow for drug loading may offer opportunities for personalized therapies in the future, incorporating ROCK inhibitors, nerve growth factors (NGF), other growth factors, or anti-inflammatory agents. Such strategies have the potential to modulate immunological processes therapeutically in the context of corneal transplantation. Moreover, advancements in storage processes and infrastructure could lead to sustainable improvements in this field, ensuring better availability and efficacy of artificial corneal endothelial replacements. One promising approach to meet these challenges is the use of biotechnological methods to fabricate standardized and scalable artificial DM implants for replacement membranes.

This overview article highlights the relevant aspects and challenges associated with fabricating artificial DM implants. Considering the current state of technology and recent developments, future advancements are likely to further shape the field. For instance, optimizing the biofabrication process for DMEK grafts to satisfy stringent requirements presents significant obstacles. The use of donor cells introduces logistical challenges, necessitating scalable fabrication processes and cost-effective solutions. Personalized approaches using patient-derived cells may provide benefits but also introduce complexities in cultivation procedures, potentially resulting in higher failure rates and increased treatment costs. Furthermore, precise sizing and orientation of grafts are essential for successful transplantation. While various imaging techniques facilitate in vivo monitoring of grafts, comprehensive biometric analytical methods are vital to ensure graft suitability. Additionally, integrating bioengineered corneal substitutes with host tissue presents a major challenge, with immunological compatibility being a key concern. Advanced imaging and diagnostic tools are essential for monitoring cell functionality and integration, providing immediate feedback for adjusting therapeutic strategies.

In conclusion, interdisciplinary approaches that combine cellular biology, materials science, and clinical practices are essential to overcoming these challenges and enhancing the success rates of DMEK procedures relying on biofabricated implants. Animal studies, such as those using murine or porcine models, are crucial for validating stability and integration before clinical implementation. Moreover, exploring regenerative medicine strategies to enhance endothelial cell survival and function post-transplantation offers promising avenues for improving outcomes in DMEK procedures. Targeted delivery systems and preconditioning techniques may play pivotal roles in creating a favorable post-transplant environment, ultimately advancing the field of corneal transplantation and regenerative medicine in ophthalmology.
